# Efficacy of different polypill combinations for primary and secondary cardiovascular disease prevention: a systematic review and meta-analysis

**DOI:** 10.3389/fcvm.2025.1558579

**Published:** 2025-06-09

**Authors:** Habib Yazgi, Shivani Mattikalli, Brian Fang, Hannah Heselton, Paddy Ssentongo, Michael Farbaniec

**Affiliations:** ^1^ Department of Medicine, Penn State Health Milton S. Hershey Medical Center, Hershey, PA, United States; ^2^ Division of Infectious Diseases, Department of Medicine, Penn State Health Milton S. Hershey Medical Center, Hershey, PA, United States; ^3^ Department of Public Health Sciences, Penn State Health Milton S. Hershey Medical Center, Hershey, PA, United States; ^4^ Division of Cardiology, Department of Medicine, Penn State Health Milton S. Hershey Medical Center, Hershey, PA, United States

**Keywords:** polypill, cardiovascular disease, major adverse cardiac events, mortality, meta-analyses

## Abstract

**Background:**

Cardiovascular disease is the leading cause of mortality and morbidity worldwide, and polypills have established efficacy in preventing poor outcomes. However, evidence on the optimal polypill combination is lacking. The objective of the study is to estimate the optimal polypill combination that maximizes cardiovascular outcomes.

**Methods:**

MEDLINE/PubMed, Scopus, and Cochrane Database of Systematic Reviews databases were searched from January 1, 1960, to March 30, 2025. Studies that provided data on the association between polypill and cardiovascular outcomes were included. We estimated the effect of various polypill combinations by random-effects meta-analyses using the generic inverse variance method. Subgroup and meta-regression analyses were conducted to explore potential effect modification in the association between polypill combinations and cardiovascular outcomes.

**Results:**

Thirty studies comprising 35,833 individuals met the inclusion criteria from 6 continents. The estimated pooled effects of polypill use on major adverse cardiovascular events (MACE), cardiovascular death, and all-cause mortality were RR 0.78 (95% CI, 0.63–0.97), 0.75 (95% CI, 0.63–0.89), 0.88 (95% CI, 0.79–0.98), respectively. The pooled relative risk of MACE outcome was 21% lower in combination of 4 or more pills [0.79 (95% CI, 0.55–1.15), *n* = 6 studies] vs. to 22% and 3 or less combination of medication classes (RR: 0.78 95% CI: 0.70–0.86), *n* = 4 studies). Polypill combinations containing moderate or high-intensity statins were associated with lower risk of MACE outcomes RR 0.79 95% CI: 0.70–0.97), *n* = 2 studies compared to combinations with low-intensity statins RR 0.78 95% CI: 0.59–1.03, *n* = 8). All polypills for MACE outcomes contained RAAS inhibitors. Calcium channel blockers, RAAS inhibitors and diuretics-containing polypills were associated with the highest reduction in blood pressure. Certainty of evidence for MACE ranged from low to high, with most trials rated as moderate to high.

**Conclusions:**

In this meta-analysis, polypills with 3 cardiovascular classes that contain a high-intensity statins, aspirin and RAAS inhibitors appeared to have greater reduction in MACE outcomes. The presence of a diuretic and a calcium channel blocker in the polypill was associated with greater reductions in systolic and diastolic blood pressure.

## 1 Introduction

Cardiovascular disease (CVD) is the leading cause of death globally, with ischemic heart disease and stroke comprising more than 80% of this group. Over the last two decades, the rate of cardiovascular disease-associated death, adjusted for age, has steadily declined, but this rate of decline has recently plateaued in high-income countries (1, 2). High low-density lipoprotein (LDL) cholesterol and high blood pressure are two significant risk factors for cardiovascular disease-associated mortality and morbidity (3, 4). Randomized trials showed that drugs to lower three risk factors—LDL cholesterol (5), blood pressure (6–8), and platelet function (with aspirin)—reduce the incidence of ischemic heart disease (IHD) events and stroke (9).

About 1 in 4 people do not adhere well to prescribed drug therapy (10). Poor adherence is considered a significant factor in treatment failure and is one of the leading challenges to healthcare professionals (11). In general, poor adherence to medications has also been linked to worse clinical outcomes (12). Non-adherence to cardiovascular medications has been associated with an increased risk of morbidity and mortality (13, 14). Further, in chronic coronary artery disease setting, non-adherence to cardioprotective medications (statins and/or angiotensin-converting enzyme inhibitors) was associated with a 10% to 40% relative increase in the risk of cardiovascular hospitalizations and a 50%–80% relative increase in the risk of mortality (15).

Polypills have been shown to improve medication adherence for CVD (16, 17). The reason behind the increased adherence to using a polypill for the prevention of CVD is that it simplifies medication intake (18). In several studies, fixed-dose polypills have been shown to be superior to usual care in reducing high blood pressure and high LDL cholesterol (19–21).

Findings from previous meta-analyses on the efficacy of the polypill have been inconsistent. Meta-analyses by Kandil, Virk, and Joseph confirm the efficacy of polypill interventions in primary prevention of cardiovascular disease (CVD) (21–23). They collectively show that polypills reduce the risk of major adverse cardiovascular events (MACE), including CVD mortality, myocardial infarction, stroke, and cardiovascular death, while also lowering systolic blood pressure (SBP) and low-density lipoprotein cholesterol (LDL-C). On the other hand, a 2022 meta-analysis did not demonstrate the benefit of polypill in reducing all-cause and CVD mortality. Additionally, the components of a polypill can vary significantly, and in some cases, some drugs, such as aspirin, are not included despite their proven efficacy in the primary and secondary prevention of cardiovascular disease and perhaps this can explain the inconsistencies in these meta-analyses (24–26).

To address the inconsistency in the aforementioned meta-analyses, we explored the effects of various medication combinations—comprising statins, multiple blood pressure–lowering drugs, and aspirin—on CVD outcomes. Our approach is distinctive in that it goes beyond the general focus on polypills in previous studies, aiming to assess a broader array of medication subsets to identify the most effective polypill composition for heart disease prevention. By methodically evaluating the additive potential of different drug combinations, our study aims to fill a literature gap, offering a novel contribution to cardiovascular disease prevention. We compare the impacts of various polypill formulations on mortality and disease outcomes from a primary and secondary prevention standpoint to provide insights on the most beneficial combinations with additive effect, guiding future research and informing clinical practice with effective preventive strategies.

## 2 Methods

### 2.1 Data sources and searches

We searched the MEDLINE/PubMed, Scopus, and Cochrane Database of Systematic Reviews databases for studies published since inception through March 30, 2025, using a combination of Medical Subject Headings (MeSH) and keywords in the title and abstract related to polypill use in cardiovascular disease prevention. We used the terms *Polypill* combined with *cardiovascular disease (e.g., myocardial infarction, stroke, blood pressure, cholesterol types) combined* with *adherence* to search peer-reviewed publications. This study was reported according to the Meta-analysis of Observational Studies in Epidemiology (MOOSE) reporting guidelines and the Preferred Reporting Items for Systematic Reviews and Meta-analyses (PRISMA) reporting guidelines (27, 28).

We also searched the reference lists of retrieved articles to identify additional relevant studies. We included studies that evaluated: (1) the effect of polypill use on medication adherence; (2) the effect of polypill use on primary cardiovascular disease prevention; and (3) the effect of polypill use on secondary cardiovascular disease outcomes. Studies not conducted in humans, case reports, letters to the editor, case series, case-control studies, practice guidelines, meta-analyses, literature reviews, and commentaries were excluded. We did not impose any restrictions based on the language of the articles or country of study. Some studies used the same population in several articles. We excluded articles with overlapping study populations.

### 2.2 Study selection

Studies were selected according to Participant (P), Intervention (I), Comparator [C], Outcome (O), and Study type (S) [PICOS] criteria (29):

**Population:** Adults aged ≥18 years with or at risk for cardiovascular disease, eligible for either primary or secondary prevention.

**Intervention:** Polypills were defined as fixed-dose combination therapy including at least two cardiovascular medications (e.g., statins, antihypertensives, aspirin).

**Comparator:** Standard of care, which included monotherapy (e.g., individual components of the polypill administered separately) or placebo.

**Outcomes:**

**Primary outcomes:**

1. **Major Adverse Cardiovascular Events (MACE):** As defined by each study, typically including composite outcomes such as cardiovascular death, myocardial infarction, stroke, or hospitalization for heart failure.
2. **Cardiovascular mortality**
3. **All-cause mortality**

**Secondary outcomes:**

1. **Change in systolic and diastolic blood pressure (mmHg)**
2. **Change in low-density lipoprotein cholesterol (LDL-C, mg/dl)**
3. **Medication adherence**, defined as the proportion of days covered (PDC), self-reported compliance, or pill counts as reported by individual studies.

**Study Design:** Randomized controlled trials and observational cohort studies.

### 2.3 Data extraction

**Figure 1:**
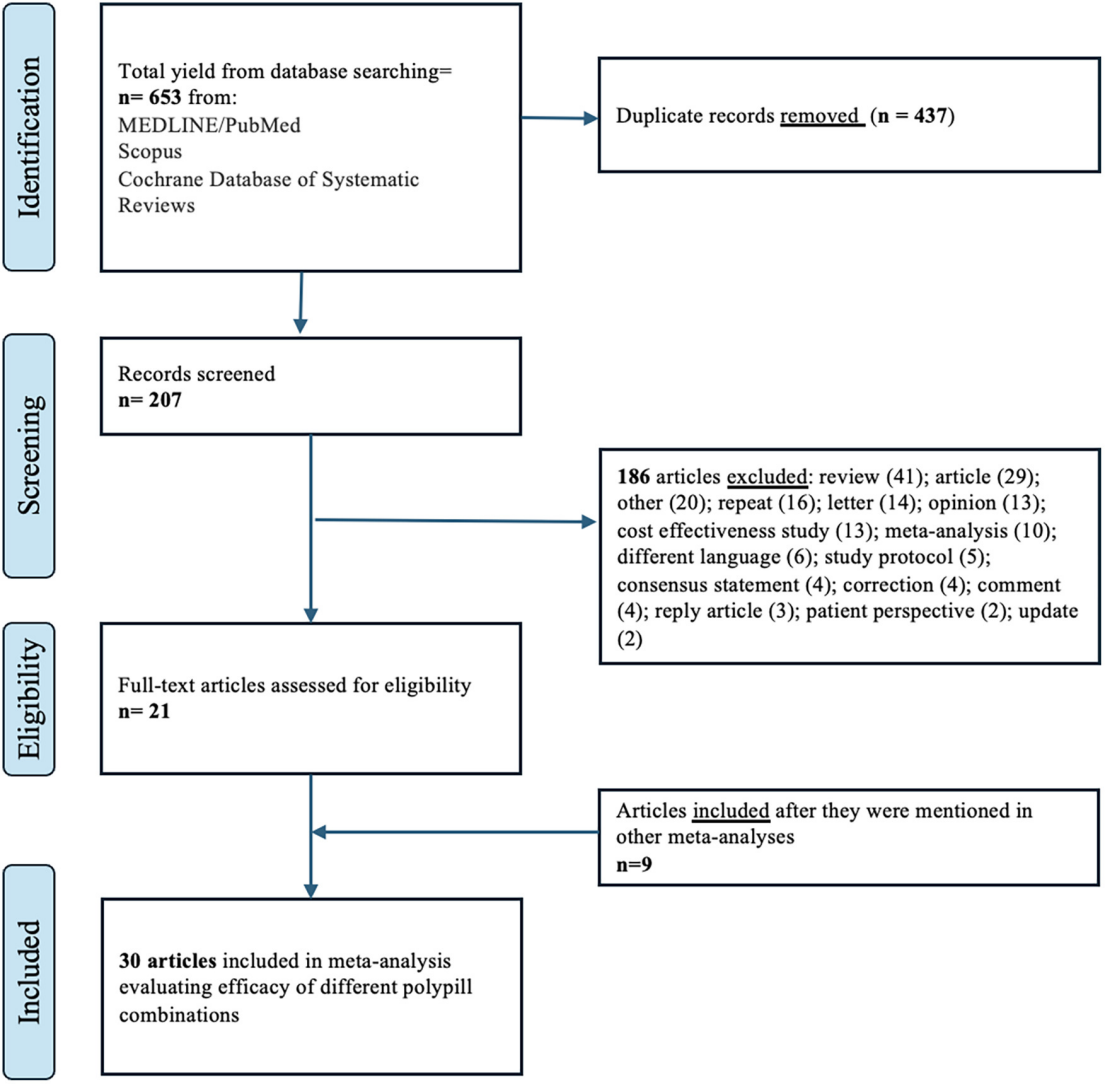
PRISMA flow diagram.

A standardized data extraction form was developed and three investigators (S.M., H.H., and H.Y) independently screened the titles and abstracts of articles, obtained the full-text articles, and performed data extraction on those meeting the inclusion criteria. Three investigators (S.M., H.H., and P.S) jointly reviewed a random subset of articles to ensure selection accuracy. Disagreements about the included articles were resolved by the senior investigator (P.S.). A detailed account of the inclusion/exclusion process is shown in Figure 1. The following information was extracted: year of study publication, country and time frame, follow-up time, study-level descriptive statistics [mean (SD)/ median (IQR) age in years, proportion (%) female, male and obese], the content of medications in each polypill, and adherence information. For primary outcomes of major adverse cardiovascular events (MACE, cardiovascular mortality and all-cause mortality, we extracted risk ratios (RR) and 95% confidence intervals or the raw values values in each group. For secondary outcomes, we extracted change in systolic and diastolic blood pressure (mmHg), change in low-density lipoprotein cholesterol (LDL-C, mg/dl) from baseline and the proportion of patients (in percentage) who were adherent to polypills.

### 2.4 Risk of bias and evidence grading

Three authors (S.M. HH and PS) independently assessed the quality of the articles included in our analysis. The risk of bias of the included RCTs was evaluated with the Cochrane Collaboration's Risk of Bias 2 tool, which evaluates five key domains: the randomization process, deviations from intended interventions, missing outcome data, measurement of outcomes, and selection of reported results (30). For nonrandomized observational studies, methodological quality was assessed with the Newcastle–Ottawa Scale (NOS) (31). As described by the NOS criteria, we assigned a maximum of 4 stars for selection, 2 stars for comparability, and 3 stars for exposure and outcome assessment. Studies with fewer than 5 stars were considered low quality; 5–7 stars, moderate quality; and more than 7 stars, high quality. Studies were included regardless of the risk of bias and quality scores, but a sub-group analysis was conducted by study type (RCT vs. non-RCT) in accordance with the framework for combining RCTs and non-RCTs (32).

To evaluate the certainty of evidence for the major adverse cardiovascular events outcome we applied the Grading of Recommendations, Assessment, Development, and Evaluations (GRADE) framework (33). RCTs were assessed across five domains: risk of bias, inconsistency, indirectness, imprecision, and publication bias. Certainty was rated as high, moderate, low, or very low.

### 2.5 Data synthesis and statistical analysis

We adopted a narrative approach describing the number of studies, study settings, and types of polypills. Descriptive statistics are reported as population proportions and medians (interquartile range). We applied random-effects models to estimate the association between polypills and primary outcomes of interest, including MACE, cardiovascular mortality, and all-cause mortality (34). We reported relative risks (RRs) and associated 95% confidence intervals. A random-effects meta-analysis was performed using the generic inverse variance method. We estimated all parameters by maximizing the pseudolikelihood. Individual and pooled estimates are displayed using forest plots. Between-study variation was assessed using *I*^2^, which describes the percentage of total variation across studies that is due to heterogeneity rather than chance, expressed as a percentage [low (25%), moderate (50%), and high (75%)] (35).

We conducted random-effects subgroup analysis and metaregression analysis to investigate the sources of heterogeneity. We examined the associations of each explanatory variable included in the metaregression associated with cardiovascular outcomes. These variables included study-level median or mean age, the proportion of males, and the year of study. Differences in risk estimates were estimated using various combinations of polypills. We also examined the effects of various polypill combinations on blood pressure and LDL cholesterol reduction.

To evaluate possible publication bias, we visually inspected the funnel plot for asymmetry by plotting the study effect size against S.E.s of the effect size (36). We performed the Egger linear regression test and the Begg rank correlation test (37). The Duval and Tweedie trim and fill procedure was used to adjust for the publication bias (38). An influence and outlier study sensitivity analysis were undertaken to estimate the association of each study with the overall pooled estimate. The *metagen* and *forest* function from the R package *meta* were used for the analysis. All statistical analyses were performed with R software, version 4.3.2 (R Foundation). The significance level was set at *P* < .05, and all *P* values were 2-tailed.

## 3 Results

### 3.1 Overview of the results

The initial literature search yielded 653 articles Figure 1; of these, we excluded 437 duplicates. After a review of titles and abstracts, we excluded 186 articles if they (1) were cost-effective studies; (2) were case series, case-control, reviews, letters, and opinions; (3) did not have a control group. We included 30 articles in this meta-analysis; 24 studies were randomized controlled trials and 6 were cohort studies. 22 studies focused on primary prevention, while 8 focused on secondary prevention. A study by Yusuf et al. was used in calculating the effect estimates in polypills with and without aspirin (25). Similarly, the study by Lafeber et al. evaluated the effect of polypills in the morning and the evening (39). Although both the PolyIran and PolyIran-Liver trials recruited participants from the Golestan Cohort Study, the trials employed distinct randomization schemes and eligibility criteria. It is therefore unlikely that the same randomized participants were included in both trials. Therefore, we included both studies in our meta-analysis.

The final studies were from 6 continents and are categorized by World Health Organization regions as follows: Africa (1 study, 3%), Asia (7 studies, 23%), Europe (7 studies, 23%), multiple continents (7 studies, 23%), North America (6 studies, 20%), Oceania (2 studies, 7%), and South America (1 study, 3%). The present analysis included a total sample of 35,833 individuals. The number of individuals included in individual studies ranged widely (78 to maximum 6,838), with a median of 477 patients (interquartile range, 207–1,270). Details of each study included in the meta-analysis are provided in Table 1. The median age of participants was 61 years (IQR 58–64) and median proportional of male sex was 61 (IQR 52–73).

**Table 1:** Study characteristics.

Authors	Publication year	Country	Study type	Age (median or mean)	Male (% total)	Sample Size	Follow-up, median month	Quality Score	MACE or other outcomes
Sarfo et al. (40)	2023	Ghana	RCT	58	49	148	12	See eTable 1	Change in CIMT over 12 months
Chávez Fernández et al. (41)	2023	Mexico	Cohort study	64	64	479	97	NOS stars 6	Change lipid profile, and blood pressure
Castellano et al. (42)	2022	Spain, Czech Republic, France, Germany, Hungary, Poland, Italy	RCT	76	69	2,499	36	See eTable 1	CVD death, nonfatal type 1 MI, nonfatal ischemic stroke, or urgent coronary revascularization
Mostaza el al. (43)	2022	Spain, Portugal, Mexico	RCT	65	60	439	4	See eTable 1	Mean change in LDL-c and SBP
Merat et al. (44)	2022	Iran	RCT		51	1,508	60	See eTable 1	MI, sudden death, new-onset HF, CARP; fatal and non-fatal stroke, or hospitalization for an acute coronary event.
Portela-Romeroa et al. (45)	2021	Spain	Cohort study	72	61	547	55	NOS stars 6	
Yusuf et al. (25)	2021	India, Philippines, Colombia, Bangladesh, Canada, Malaysia, Tunisia, Indonesia, Tanzania	RCT	64	46	5,713	55	See eTable 1	CVD death, stroke, MI, HF, resuscitated cardiac arrest, and CARP
Castello et al. (46)	2020	Spain	Cohort study	70	61	104	3	NOS stars 7	Stroke recurrence, reduction in BP, reduction in LDL cholesterol
Gómez-Álvarez et al. (47)	2020	Mexico	Cohort study	57	53	533	12	NOS stars 7	Lipid profile
González-Juanatey et al. (48)	2019	USA	RCT	55	58	241	2	See eTable 1	BP (ambulatory BP measurement) and LDL-c
Munoz et al. (16)	2019	USA	RCT	56	44	303	12	See eTable 1	SBP and LDL-c
Roshandel et al. (49)	2019	Iran	RCT	59	49	6,838	2	See eTable 1	Hospitalization for ACS, fatal MI, sudden death, HF, CARP, and non-fatal and fatal stroke
Lafeber et al. (50)	2017	UK, Ireland, Netherlands, India	RCT	62	82	2,004	12	See eTable 1	LDL-c and SBP
Selak et al. (51)	2014	New Zealand	RCT	62	64	513	24	See eTable 1	Adherence to medications, mean change in BP and LDL-c
Lafeber et al. (39)	2016	Australia, Brazil, India, The Netherlands, New Zealand, UK, USA	RCT	62	81	378	3	See eTable 1	Change in LDL-c, SBP and adverse events
Yusuf et al. (52)	2012	India	RCT	58	59	514	2	See eTable 1	Change in BP, heart rate, Lipids, urinary K+, and tolerability
Rogers et al. (53)	2011	Australia, Brazil, India, Netherlands, New Zealand, US	RCT	61	81	378	3	See eTable 1	Change in SBP, LDL-c and tolerability of medications
Soliman et al. (54)	2011	Sri Lanka	RCT	59	24	203	3	See eTable 1	Change in SPB, total cholesterol and estimated 10-year CVD risk
Patel et al. (55)	2015	Australia	RCT	64	63	623	19	See eTable 2	Medication adherence, SBP and total cholesterol
Malekzadeh et al. (56)	2010	Iran	RCT	59	62	475	12	See eTable 1	Change in LDL-c, SBP, DBP and adverse reactions
Yusuf et al (57)	2009	India	RCT	54	56	2,053	3	See eTable 1	LDL-c, BP, HR, urinary 11-dehydrothromboxane, medication adherence and safety
Zamorano et al. (58)	2011	Argentina, Brazil, China, Egypt, France, Germany, Hungary, India, Italy, Korea, Mexico, Philippines, Poland, Russia, South Africa, Spain, Turkey, United Arab Emirates, and Venezuela	RCT	60	52	1,461	12	See eTable 1	Framingham 10-year CHD risk. BP was also studied
Lafeber et al. (59)	2015	The Netherlands	RCT	67	86	78	2	See eTable 1	Morning vs evening effect of polypill on LDL-c and SBP
Grimm et al. (60)	2010	USA	RCT	56	54	218	1.5	See eTable 1	Change in BP and LDL-c
Mariani et al. (61)	2020	Argentina	RCT	54	90	100	6	See eTable 1	Adherence. Also studied were mean change in HR, cholesterol CRP, and platelet aggregation.
Neutel et al. (62)	2009	USA	RCT	53	54	123	1.5	See eTable 1	Mean change in BP, and LDL-c
Gonzalez- Juantely et al. (63)	2022	Spain	Cohort study			6,456	24	NOS stars 8	IHD, CVD and PVD
Castellano et al. (17)	2014	Argentina, Italy, Paraguay, Spain	RCT			695	9	See eTable 1	Adherence to medication
Oh et al. (64)	2018	Korea	RCT	81	81	125	2	See eTable 1	Change in mean in SBP, DPB LDL-C
Wald et al. (65)	2012	UK	RCT	59	74	84	3	See eTable 1	Mean change in BP and LDL-c

Abbreviated: RCT, randomized controlled trials; MI, myocardial infarction; CVD, cardiovascular disease; PVD, peripheral vascular disease; IHD, ischemic heart disease; CIMT, carotid intima-media thickness; ACS, acute coronary syndrome; CARP, coronary artery revascularization procedures; SBP, systolic blood pressure; DBP, diastolic blood pressure; LDL-c, low-density lipoprotein cholesterol; HR, heart rate; CRP, C-reactive protein; CHD, coronary heart disease; NOS, Newcastle-Ottawa Scale; MACE, major adverse cardiovascular events; USA, United States of America; UK, United Kingdom.

### 3.2 The association of polypill use and major adverse cardiovascular events

**Figure 2:**
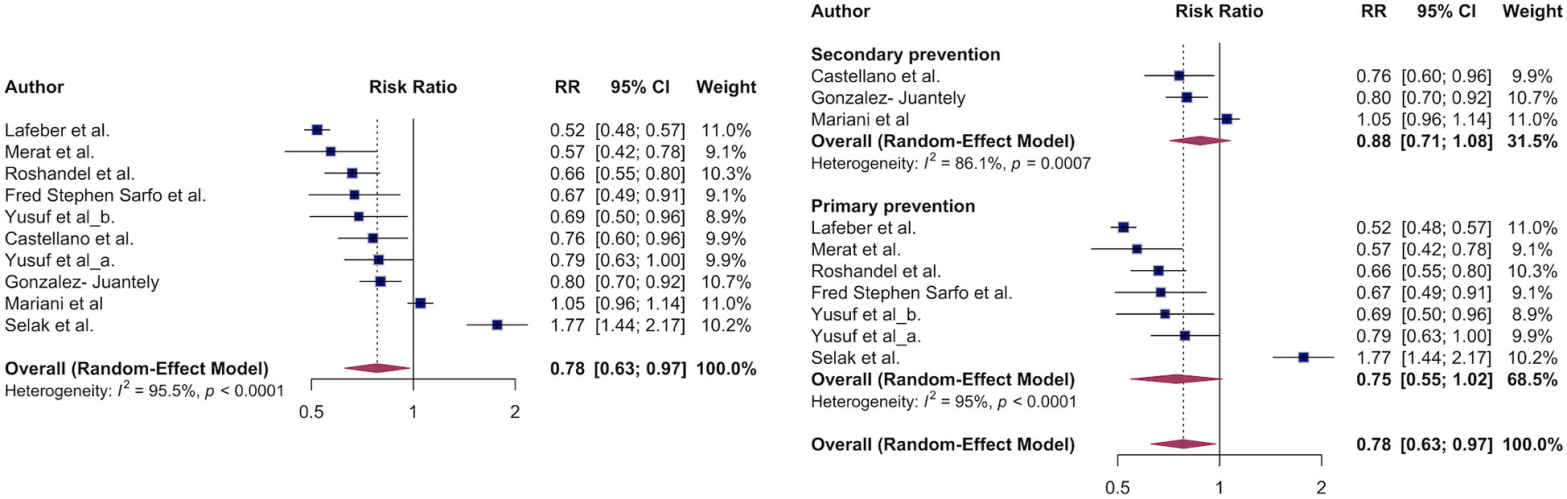
The association of major adverse cardiovascular events and polypill. Overall survival benefit of polypill on MACE outcomes **(A)**. Polypill effect on MACE stratified by primary vs. secondary CVD prevention.

First, we estimated the effect of polypill on major adverse cardiovascular events. Compared to individual agents, polypills were associated with a 22% lower risk of major adverse cardiovascular events: RR: 0.78 (95% CI, 0.63–0.97, *I*^2^ ^=^ 96%) (Figure 2A). When stratified by primary vs. secondary prevention, there was no significant difference in effect estimates: RR: 0.75 (95% CI, 0.55–1.02; *I*² = 95%) for primary prevention and RR: 0.88 (95% CI, 0.71–1.08; *I*² = 86%) for secondary prevention (*p* for subgroup difference = 0.40) (Figure 2B). All studies except one were RCTs and sensitivity analysis of comparing RCT vs. non-RCT did not cause meaningful differences in the effect estimates RR: 0.78 (95% CI, 0.61–1.00, *I*^2^ ^=^ 96%) vs. RR: 0.80(95% CI, 0.70–0.92) (Supplementary Figure S1). In all included studies that estimated MACE outcomes, the polypills evaluated contained either an angiotensin receptor blocker (ARB) or an angiotensin-converting enzyme (ACE) inhibitor.

Due to significant heterogeneity in the overall effect estimate, various subgroup analyses were conducted as summarized below:

#### 3.2.1 Aspirin

**Figure 3:**
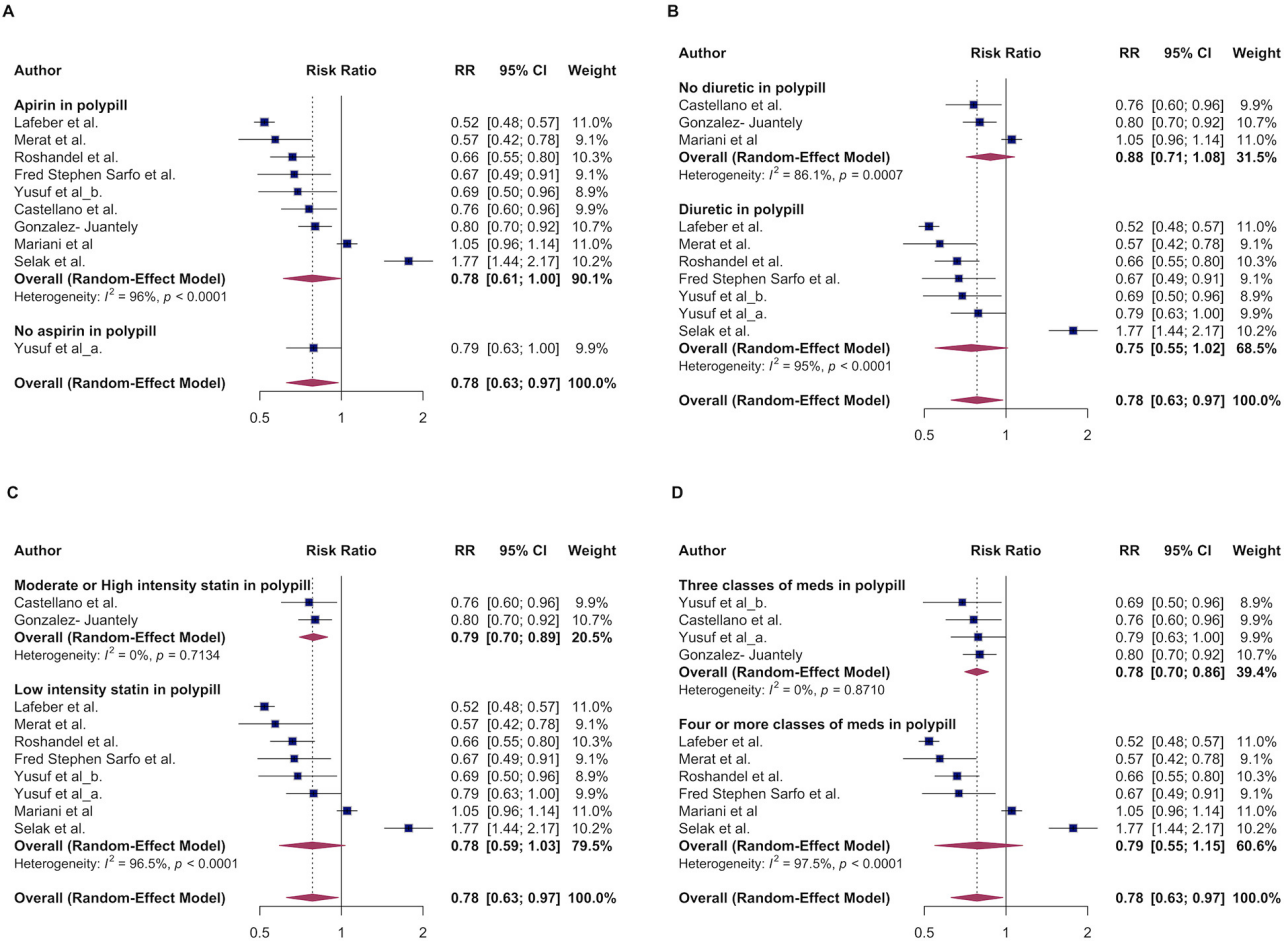
The association of major adverse cardiovascular events and polypill stratified by: aspirin-containing vs non-aspirin containing polypill **(A)**; Diuretic vs lack of diuretic in a polypill **(B)**. High/moderate intensity vs. low intensity statin in a polypill **(C)**, three or fewer vs. more than three classes of medications in a polypill **(D)**.

Next, we evaluated efficacy of aspirin-containing polypills on cardiovascular disease (CVD) primary outcomes by conducting subgroup analysis by comparing MACE outcomes between aspirin-containing polypill vs. polypills without aspirin. Most of studies analyzed, except one, included aspirin in the polypill. There was no between-group difference in the effect estimates: (RR 0.78, 95% CI: 0.61–1.00, *I*^2^ ^=^ 96%, for aspirin containing polypill) vs. RR of 0.79 (95% CI: 0.63–1.00, *I*^2^ ^=^ NA) for polypills without aspirin (Figure 3A).

#### 3.2.2 Diuretics

Next, we explored whether adding a diuretic to the poly-pill is associated with better MACE outcomes compared to the non-diuretic containing poly-pill. The pooled RR for the diuretic group was 0.75 (95% CI: 0.55–1.02, *I²* = 95%), compared to poly-pill without diuretic, RR of 0.88 (95% CI: 0.71–1.08 *I²* = 86) (Figure 3B) indicating a lack of between-group difference in MACE outcomes.

#### 3.2.3 Number of pills per fixed dose combination

We conducted various permutations of the number of components in the fixed-dose polypill to identify the optimal combination that confers maximum benefit. Of note majority of polypills combinations included 3 and more pills. We therefore compared MACE outcomes by having 4 or more vs. 3 or less medications in a fixed-dose combination. Studies with four or more pills in combination, the pooled RR estimate was 0.79, 95% CI (0.55–1.15, *I²* = 98%) suggesting a potential benefit but not statistically significant (Figure 3C). For the studies with 3 pills in combination, the pooled risk ratio was 078 (95% CI: 0.70- 0.86, *I²* = 0%), indicating significant reduction in risk. The low heterogeneity (*I*² = 0%) suggests more consistent results among these studies. In summary, the analysis of permutation of the pills number suggests that the most benefits are observed with when a fixed-dose strategy has on average three pills.

#### 3.2.4 Statins: high/moderate intensity vs. low intensity

Furthermore, polypills were analyzed based on their statin component. The fixed-dose polypills containing high- or moderate-intensity statins were compared to those with low-intensity statins, with the results highlighting differential impacts on primary MACE outcomes. Polypills with low-intensity statins demonstrated a 22% reduction in MACE outcomes (RR 0.78; 95% CI: 0.59–1.03) (Figure 3D), suggesting a lack of association. The high heterogeneity (*I²* = 97%) in this group indicates significant variability among the included studies. In contrast, polypills containing high- or moderate-intensity statins demonstrated a significant reduction in cardiovascular events, with an RR 0.79 (95% CI: 0.70–0.86), and low heterogeneity (*I²* = 0%), suggesting more consistent findings and statistical significance.

#### 3.2.5 Geographic location of studies

The analysis of polypill effects on MACE outcomes across different continents reveals varied efficacy. The one study from Africa demonstrated a significant reduction in risk (RR 0.67, 95% CI: 0.49–0.91). In Europe, the pooled effect estimate indicated a significant reduction in MACE outcomes (RR 0.79, 95% CI: 0.70–0.89) with low heterogeneity (*I²* = 0%), indicating consistent findings. Studies from Asia presented the most significant benefit with a risk ratio of 0.63 (95% CI: 0.54–0.74) (Supplementary Material 2) and low heterogeneity. Conversely, studies spanning multiple continents demonstrated a significant reduction (RR 0.72, 95% CI: 0.54–0.94) but with high heterogeneity (*I²* = 94%), suggesting large variations in the outcomes. The one study from Oceania indicated an increased risk of MACE (RR 1.77, 95% CI: 1.44–2.17), while the study from South America showed no significant effect (RR 1.05, 95% CI: 0.96–1.14). Overall, the polypill demonstrated a significant benefit in reducing MACE outcomes, with the greatest reduction observed in studies conducted in Asia.

#### 3.2.6 Meta-regression

To explore further potential sources of heterogeneity in our meta-analysis, we conducted meta-regression analyses using study-level covariates, including year of publication, mean or median age of participants, and the proportion of male participants. None of these covariates significantly influenced the pooled effect size. Specifically, meta-regression yielded a relative risk of 0.95 (95% CI, 0.88–1.02) for year of publication, 0.99 (95% CI, 0.95–1.04) for age, and 1.00 (95% CI, 0.99–1.02) for the proportion of men enrolled.

### 3.3 The association of polypill and primary CVD mortality

**Figure 4:**
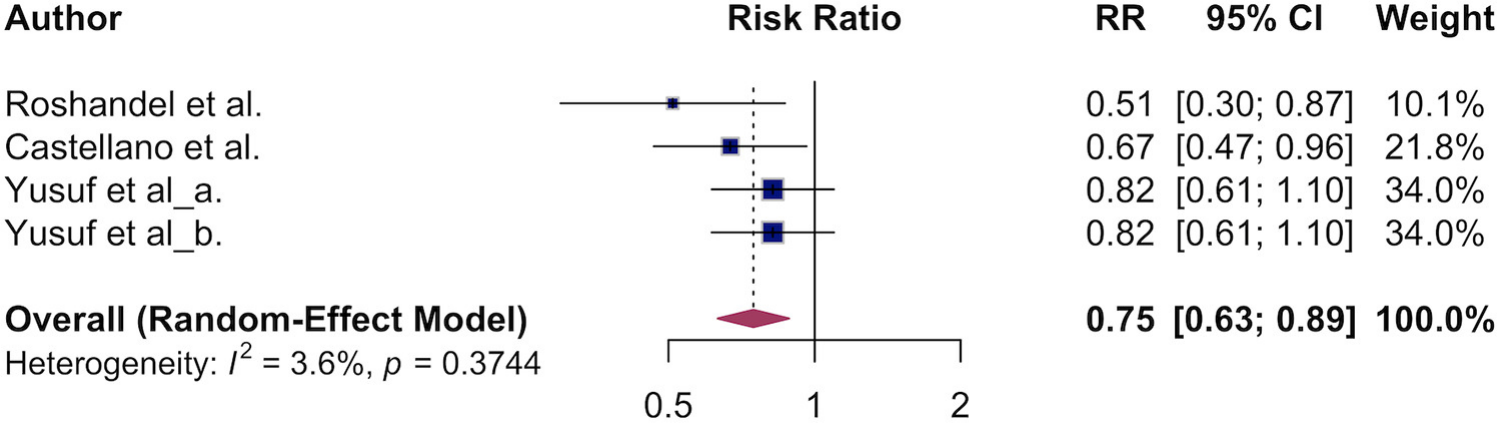
The association of polypill and primary CVD mortality.

The estimated pooled overall survival benefit of the polypill for cardiovascular death was 0.75 (95% CI, 0.63–0.89, Figure 4) translating to a 25% lower CVD mortality in the polypill vs. usual care. Low heterogeneity was observed (*I²* = 0%), indicating consistent findings across studies. Due to low variation across studies and the limited number of studies pooled for CVD mortality, we were unable to conduct a meaningful subgroup analysis.

### 3.4 Association of polypill and all-cause mortality

**Figure 5:**
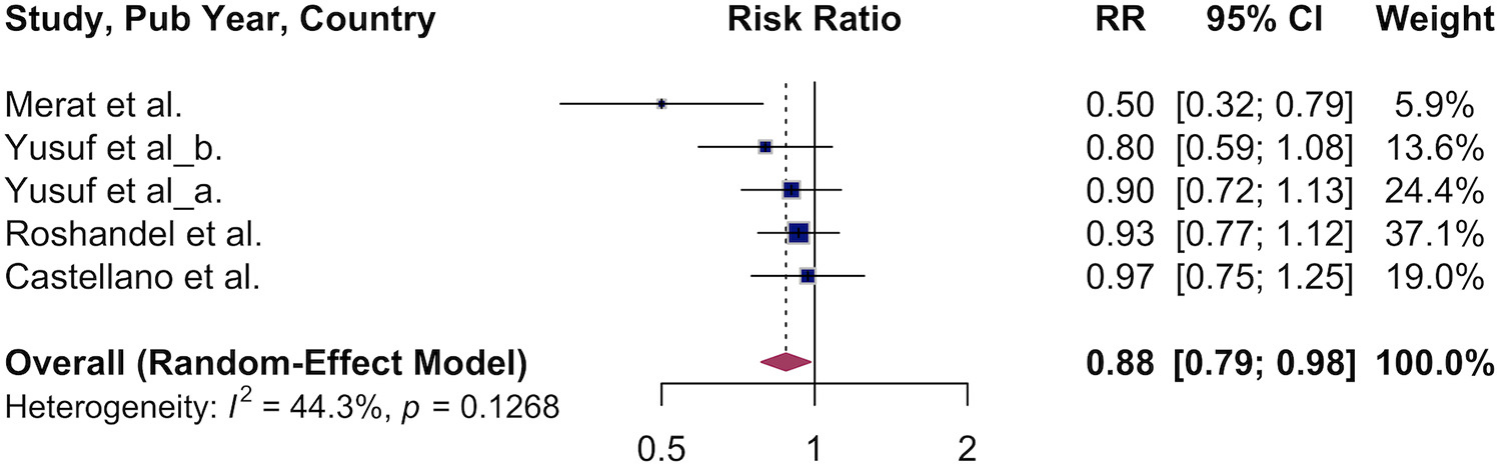
Association of polypill and all-cause mortality.

Next, we pooled the results of studies that examined the effect of polypill use vs. usual care on all-cause mortality. We observed a 12% lower overall survival in the polypill group: RR 0.88 (95% CI, 0.79–0.98, Figure 5). Variation between studies was moderate (*I*² = 44%). Due to the small number of studies, subgroup analyses were not conducted.

### 3.5 Polypills use and mean change in blood pressure

**Figure 6:**
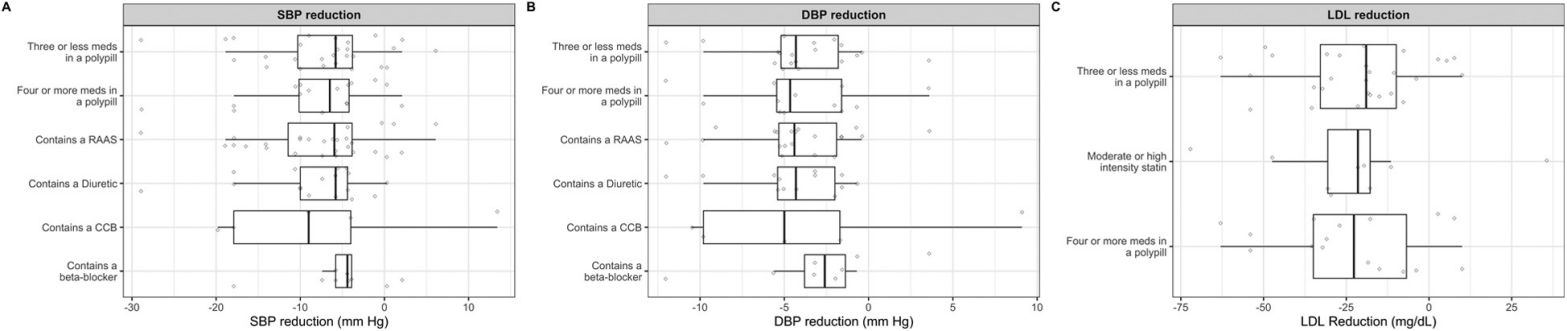
Effect of various combinations of polypills on mean change of systolic blood pressure **(A)**, diastolic blood pressure **(B)** and low-density lipoprotein cholesterol **(D)**.

Across polypill trials with available data, treatment with a cardiovascular polypill was associated with overall reductions in blood pressure and LDL cholesterol. The mean reduction in systolic blood pressure (SBP) was −7.38 mmHg in the polypill group compared to −4.65 mmHg with standard care, corresponding to a net benefit of SBP reduction of 2.72 mmHg. For diastolic blood pressure (DBP), the polypill achieved a mean reduction of −3.79 mmHg, vs. −1.97 mmHg with control, corresponding to a net benefit of 1.82 mmHg. However, the strongest systolic reduction was observed in the polypill combination that contained a calcium channel blocker with a median reduction of 9.0 mm Hg systolic blood pressure (Figure 6A) and 5.0 mm Hg diastolic blood pressure (Figure 6B). Polypills containing a diuretic and a RAAS inhibitor were also associated with a substantial reduction in diastolic blood pressure (mean of 4 mm Hg).

### 3.6 Association between polypills and LDL reduction

Regarding lipid lowering, the mean LDL-C reduction was −23.07 mg/dl in the polypill group compared to −11.0 mg/dl in the control group, resulting in a greater LDL-C lowering of approximately 12.0 mg/dl with polypill therapy. Across the different polypill combinations, the effect on low density lipoprotein cholesterol reduction varied. Polypills containing an average of three or fewer medications, four or more medications, and moderate- or high-intensity statins were associated with median LDL cholesterol reductions of 19, 22 and 22 mg/dl, respectively (Figure 6C).

### 3.7 Correlation between polypills and adherence

The mean adherence to polypill vs. usual care was 85% and 79% respectively. The correlation analysis between adherence to polypills and health outcomes evaluated three key metrics: MACE outcomes, CVD deaths and all-cause mortality. No significant correlation was observed between polypill use and CVD deaths, MACE, or all-cause mortality (Supplementary Figure S3).

### 3.8 Publication bias and certainty of evidence

Due to an adequate number of studies, publication bias was assessed only for MACE outcomes. Visual inspection of a funnel plot of the included studies did not indicate asymmetry indicative of lack of publication bias (Supplementary Figure S4). Egger's test for publication bias (*p* = 0.85) and Begg's test were non-significant (*p* = 0.39). To identify outlier studies, we further performed influence sensitivity analyses by excluding and replacing one study at a time (Leave-One-Out method) from the meta-analysis and calculated the RR for the remaining studies. No substantial change in any of the pooled RRs was observed when individual studies were removed in turn, indicating that no single study had a considerable influence on the overall pooled estimate (Supplementary Figure S5). The overall certainty of evidence for MACE ranged from low to high across studies, with the majority rated as moderate to high; full GRADE assessments are provided in Supplementary Table S2.

## 4 Discussion

In this meta-analysis of 30 studies including over 30,000 participants, we demonstrated that polypills are effective in reducing the risk of major adverse cardiovascular events MACE, cardiovascular mortality, and all-cause mortality by 22%, 25%, and 12%, respectively. The optimal pills that showed the greatest effect in reducing MACE outcomes contained 3 cardiovascular classes of medications that contain a high-intensity statins, aspirin and RAAS inhibitors. The presence of a diuretic, calcium channel blocker, and RAAS inhibitor in the polypill was associated with greater reductions in both systolic and diastolic blood pressure.

Previous meta-analyses included fewer studies and smaller sample sizes (66, 67). Bahiru et al. analyzed data from 9,059 patients and focused on the primary prevention of cardiovascular events, showing that polypills improved blood pressure and cholesterol control but did not significantly impact all-cause mortality or atherosclerotic cardiovascular disease (ASCVD) events. However, we included 30 studies with a combined sample size of over 30,000 patients, providing a more robust and comprehensive analysis. This larger dataset allows for more reliable and generalizable conclusions. Additionally, we evaluated both primary and secondary prevention, demonstrating that polypills effectively reduce cardiovascular outcomes in both contexts. Prior analyses did not break down the effects of individual components within the polypills including recent meta-analysis (68). Conversely, we examined in detail different polypill combinations, evaluating the additive effects of various medications. This approach revealed that polypills containing a high-intensity statins, RAAS inhibitors and potentially aspirin provided the most significant reductions in MACE outcomes.

By examining the additive effects of different polypill components, we identified the most effective combinations and the optimal number of medications in the polypill needed to reduce cardiovascular events. Polypills with multiple components can simultaneously target various cardiovascular risk factors. For instance, a polypill might include a statin for cholesterol management, an antihypertensive for blood pressure control, and an antiplatelet agent like aspirin for reducing clot formation. The combined effects of different classes of medications can provide additive benefits. Statins and antihypertensives together may produce greater reductions in MACE than either medication alone (69).

While improved adherence is a well-recognized benefit of polypill use, our analysis did not find a significant correlation between adherence and cardiovascular outcomes. This indicates that the benefits of polypills may extend beyond merely improving adherence. The additional benefits could be due to the pharmacodynamic interactions between the drugs, leading to more effective control of risk factors such as blood pressure and cholesterol levels.

The findings of our meta-analysis have significant implications for public health and clinical practice. Polypills should be considered for broader use in primary and secondary cardiovascular disease prevention. Pharmaceutical companies should be encouraged to develop and manufacture polypill combinations that include aspirin, diuretics, and statins, as these have shown the most promise in reducing cardiovascular events. From our analysis, 3 classes of cardiovascular medications in a pill were also associated with increased efficacy in reduced MACE outcomes. Our meta-analysis provides an opportunity for further research and optimization of polypill strategies, informing future approaches to preventative cardiovascular therapy.

## 5 Strengths and limitations

One of the strengths of our study is the significantly large dataset, including more than 30, 000 patients overall. The larger sample size and rigorous methodology ensure more reliable and generalizable results. Second, our detailed and in-depth component analysis provides new insights into the most effective polypill combinations, and what would be the optimal way to design one to target cardiovascular primary outcomes as well as blood pressure and LDL cholesterol. Third, we included primary and secondary prevention in our meta-analysis which makes data more applicable to primary and secondary prevention. Finally, to further broaden on generalizability of our findings, our meta-analysis included patients from around the world, representing diverse countries and racial backgrounds, and included both sexes, which makes it more holistic and applicable to various populations regardless of sex and race/ethnicity. However, the findings from the present meta-analysis should be interpreted considering some limitations. The included studies varied significantly in their design, populations, and methodologies. This heterogeneity can introduce variability in the results and make it challenging to draw unified conclusions. Additionally, adherence was self-reported, and no specific objective measures were used to accurately assess adherence to usual care vs. polypill therapy, which may have influenced the reported adherence rates. Also, we conducted subgroup analysis of primary from secondary prevention in MACE outcomes, we did not have sufficient papers in each group to perform a detailed stratified analysis by type of prevention—it has been known that there are some differences when it comes to secondary compared to primary and importance of cardiovascular preventative medications such as aspirin that is indicated more in secondary vs. primary. So, it would be appropriate for a future project to conduct a detailed analysis between primary and secondary prevention to observe any major differences.

## 6 Conclusions

In this meta-analysis, polypills with 3 cardiovascular classes that contain a high-intensity statins, aspirin and RAAS inhibitors appeared to have greater reduction in MACE outcomes. The presence of a diuretic, a RAAS inhibitor and a calcium channel blocker in the polypill was associated with greater reductions in systolic and diastolic blood pressure.

## Data Availability

The datasets presented in this study can be found in online repositories. The data can be found here: https://github.com/ssentongojeddy/Polypill-CVDMeta-analyis.
