# Supplementary material for: Efficacy of different polypill combinations for primary and secondary cardiovascular disease prevention: a systematic review and meta-analysis
Source: Front Cardiovasc Med. 2025 Jun 9;12:1558579. doi: 10.3389/fcvm.2025.1558579 (PMC12183251; doi:10.3389/fcvm.2025.1558579)
Supplement: Supplementary file 1 [file Datasheet1.pdf]

Efficacy of Different Polypill Combinations for Primary and Secondary Cardiovascular Disease Prevention: A Systematic Review and Meta-analysis

Habib Yazgi MD[1¶], Shivani Mattikalli BS[1¶], Brian Fang MD[1], Hannah Heselton BS[1], Paddy Ssentongo, MD, PhD [2,3]*§, Michael Farbaniec MD[4]*

[1]Department of Medicine, Penn State Health Milton S. Hershey Medical Center, Hershey, Pennsylvania, USA

[2]Division of infectious diseases, Department of Medicine, Penn State Health Milton S. Hershey Medical Center, Hershey, Pennsylvania, USA

[3]Department of Public Health Sciences, Penn State Health Milton S. Hershey Medical Center, Hershey, Pennsylvania, USA

[4]Division of Cardiology, Department of Medicine, Penn State Health Milton S. Hershey Medical Center, Hershey, Pennsylvania, USA

¶ Contributed equally as co-first authors

* Contributed equally as co-senior authors

§To whom correspondence should be addressed:
Paddy Ssentongo, MD, PhD, MPH

Division of infectious diseases and epidemiology

Department of Medicine

Penn State Hershey Medical Center

The Pennsylvania State University

Hershey, PA 17033

United States of America.

Email: pssentongo@pennstatehealth.psu.edu

**Study**	**Randomization Process**	**Deviations from Intended Interventions**	**Missing Outcome Data**	**Measurement of Outcome**	**Selection of Reported Result**	**Overall Risk**
Castellano et al. (2022)	Low	Low	Low	Low	Low	Low
Sarfo et al. (2023)	Low	Low	Some concerns	Low	Low	Some concerns
Castellano et al. (2014)	Low	Some concerns	Low	Some concerns	Low	Some concerns
Chul Oh et al. (2018)	Unclear	Unclear	Unclear	Unclear	Unclear	Unable to assess
Study	Randomization Process	Deviations from Intended Interventions	Missing Outcome Data	Measurement of Outcome	Selection of Reported Result	Overall Risk
Study	Randomization Process	Deviations from Intended Interventions	Missing Outcome Data	Measurement of Outcome	Selection of Reported Result	Overall Risk
Grimm et al. (2010)	Some concerns – Details of allocation concealment not provided; one randomized patient not treated.	Low – ITT analysis conducted, minimal deviations reported.	Low – Minimal missing data; final status obtained for nearly all participants.	Low – Objective outcomes (e.g., BP, LDL) measured via standard clinical protocols.	Low – Outcomes appear consistent with trial objectives.	Low – No major concerns across domains.
Lafeber et al. (2015, crossover)	Low – Randomized crossover design with clear sequence.	Low – Adherence to allocated timing of intervention arms.	Low – Minimal loss to follow-up, complete data available across crossover periods.	Low – Objective metrics (BP, LDL) used.	Low – All pre-specified outcomes reported.	Low – High-quality crossover RCT.
Lafeber et al.	Low – Randomized	Low – No deviations	Low – High	Low – Lab-based and	Low – Consistent	Low – No notable bias

(2016)	placebo-controlled design.	reported.	retention and analysis rate.	validated measures used.	with trial registration and objectives.	identified.
Mariani et al. (2020)	Low – Patients randomized early after MI.	Some concerns – Study was stopped early for futility.	Low – Near complete follow-up with imputation for one case.	Low – Clinical measures used objectively.	Some concerns – Early stopping may influence outcome reliability.	Some concerns – Premature termination introduces uncertainty.
Merat et al. (2022)	Some concerns – No allocation concealment; open-label design.	Low – Prespecified interventions followed.	Some concerns – Greater dropout in control arm at final visit.	Low – Outcomes measured independently by GCS follow-up team.	Low – Reported as planned in analysis.	Some concerns – Due to open-label design and selective dropout.
Muñoz et al. (2019)	Low – Proper randomization; baseline balance achieved.	Low – High adherence to polypill (86%); minimal deviations.	Low – Very low attrition rate.	Low – Objective clinical outcomes (BP, LDL) used.	Low – Outcomes consistent with pre-specified primary endpoints.	Low – No notable risk across domains.
Neutel et al. (2009)	Low – Proper randomization and concealment reported.	Low – Protocol adhered to; crossover allowed per protocol.	Low – High follow-up rate at 8 weeks.	Low – Objective clinical endpoints (BP, LDL-C).	Low – Reported as per protocol.	Low – High-quality short-term RCT.
Patel et al. (2015) [Kanyini GAP]	Low – Central randomization with stratification.	Low – Open-label but minimal deviation reported.	Low – Primary outcome reported in most participants.	Low – Objective self-report adherence, BP and cholesterol measured.	Low – Primary outcomes consistent with protocol.	Low – Well-designed pragmatic trial.
Portela-Romero et al. (2021)	Not an RCT – Observational design with pre-post analysis.	Not an RCT	Low – Reported data on 547 patients.	Low – Standard measures for LDL and BP.	Low – Pre-specified comparison groups.	Not an RCT
Rogers et al. (2011) [PILL trial]	Low – Double-blind, placebo-controlled with centralized randomization.	Some concerns – High early discontinuation in polypill group (23%).	Low – Outcomes reported for all randomized	Low – Objectively measured BP and LDL.	Low – Primary outcomes predefined and consistentl	Some concerns – Early discontinuation affects interpretation.

			participants.		y reported.	
Yusuf et al. (2021) [TIPS-3]	Low – Central computerized system with stratification by center.	Low – High adherence during run-in; flexibility allowed for symptomatic participants.	Low – 5713 randomized with mean 4.6 years follow-up; high retention.	Low – Objectively measured outcomes (CV death, MI, stroke).	Low – All outcomes pre-specified and reported in NEJM trial registry.	Low – Robust factorial RCT with transparent reporting and high methodological rigor.
Yusuf et al. (2009) [TIPS-1]	Low – Central secure website used; multicenter India trial.	Low – Blinded capsule administration ensured protocol adherence.	Low – Most patients completed 12-week active drug phase; missingness low.	Low – BP, LDL, and biomarkers objectively measured with validated methods.	Low – Pre-specified outcomes clearly stated and analyzed.	Low – Double-blind RCT focused on intermediate endpoints with full transparency.
Yusuf et al. (2012) [TIPS-2]	Low – Central computerized allocation with post-randomization blinding intact.	Some concerns – Potassium arm misassigned due to programming error.	Low – Data available for >90% for primary outcomes.	Low – Central lab measurements for LDL, BP, K+, etc.	Low – Primary analyses pre-specified and consistently reported.	Some concerns – Due to open-label potassium co-intervention allocation error.

Supplementary Table 1: Cochrane-risk-of-bias tool for randomized trials

**Study**	**Risk of Bias**	**Inconsistency**	**Indirectness**	**Imprecision**	**Publication Bias**	**Overall Certainty**
Castellano et al. (2022) [SECURE]	Low	No serious inconsistency	No serious indirectness	No serious imprecision	Undetected	High
Sarfo et al. (2023) [SMAART]	Some concerns	Serious	Serious	Serious	Undetected	Low

González-Juanatey et al. (2022) [NEPTUNO]	Moderate	No serious inconsistency	No serious indirectness	No serious imprecision	Undetected	Moderate
Lafeber et al. (2016)	Low	Serious	No serious indirectness	No serious imprecision	Undetected	Moderate
Mariani et al. (2020)	Some concerns	Serious	No serious indirectness	Serious	Undetected	Low
Merat et al. (2022) [PolyIran-Liver]	Low	No serious inconsistency	No serious indirectness	No serious imprecision	Undetected	High
Roshandel et al. (2019) [PolyIran]	Low	No serious inconsistency	No serious indirectness	No serious imprecision	Undetected	High
Selak et al. (2014) [IMPACT]	Some concerns	No serious inconsistency	No serious indirectness	No serious imprecision	Undetected	Moderate
Yusuf et al. (2021) [TIPS-3]	Low	No serious inconsistency	No serious indirectness	No serious imprecision	Undetected	High

Supplementary Table 2: GRADE Summary for MACE Outcome

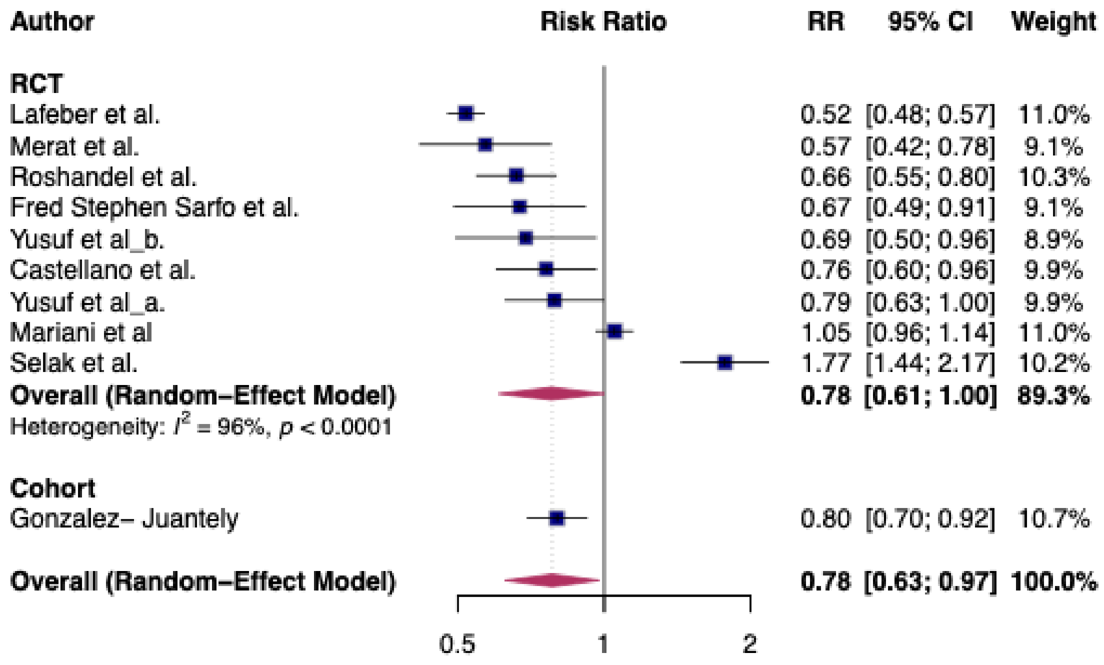

Supplement figure 1: Comparison of MACE effect estimates between RCTs vs Cohort studies.

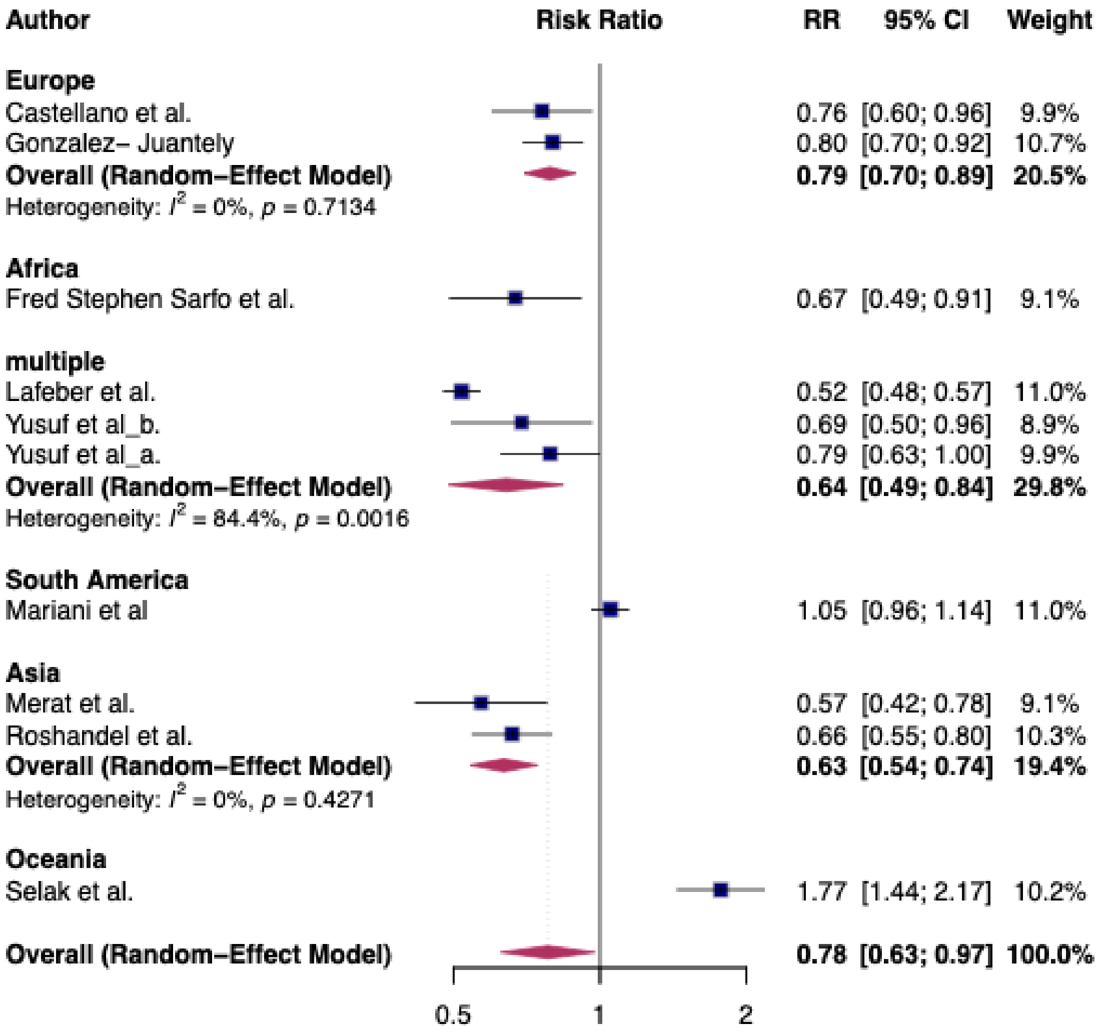

Supplement figure 2: Polypill effect on primary MACE, stratified by continent.

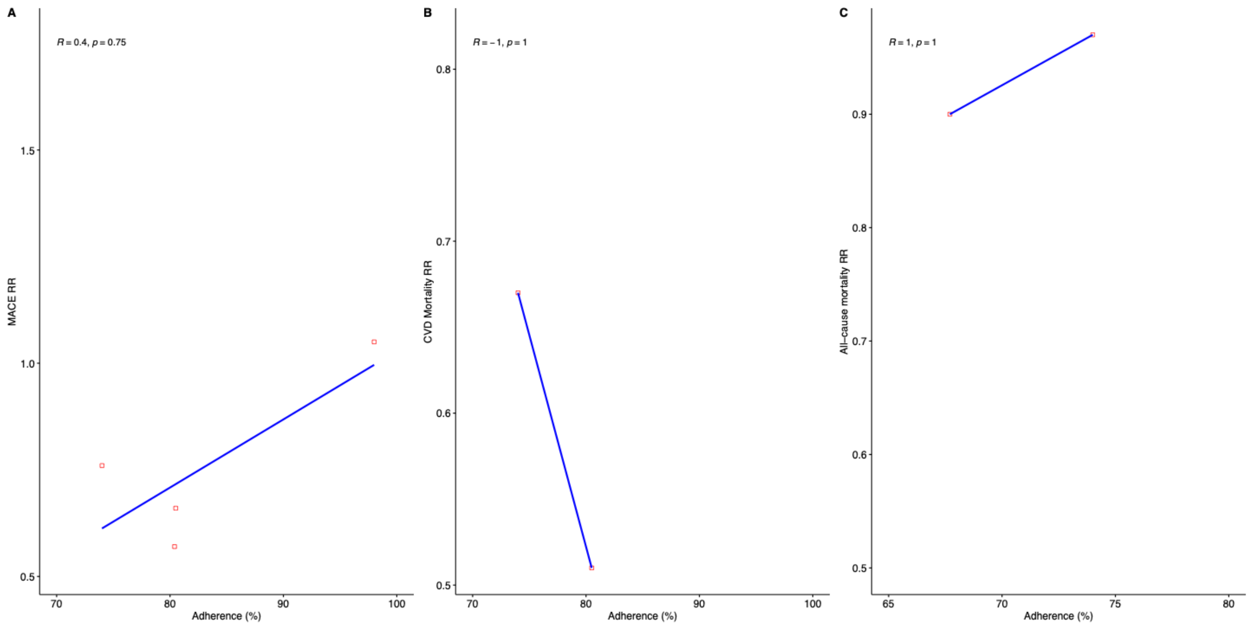

Supplement figure 3: **Correlation between polypills and adherence: MACE (A), CVD mortality (B); All-cause mortality (D)**

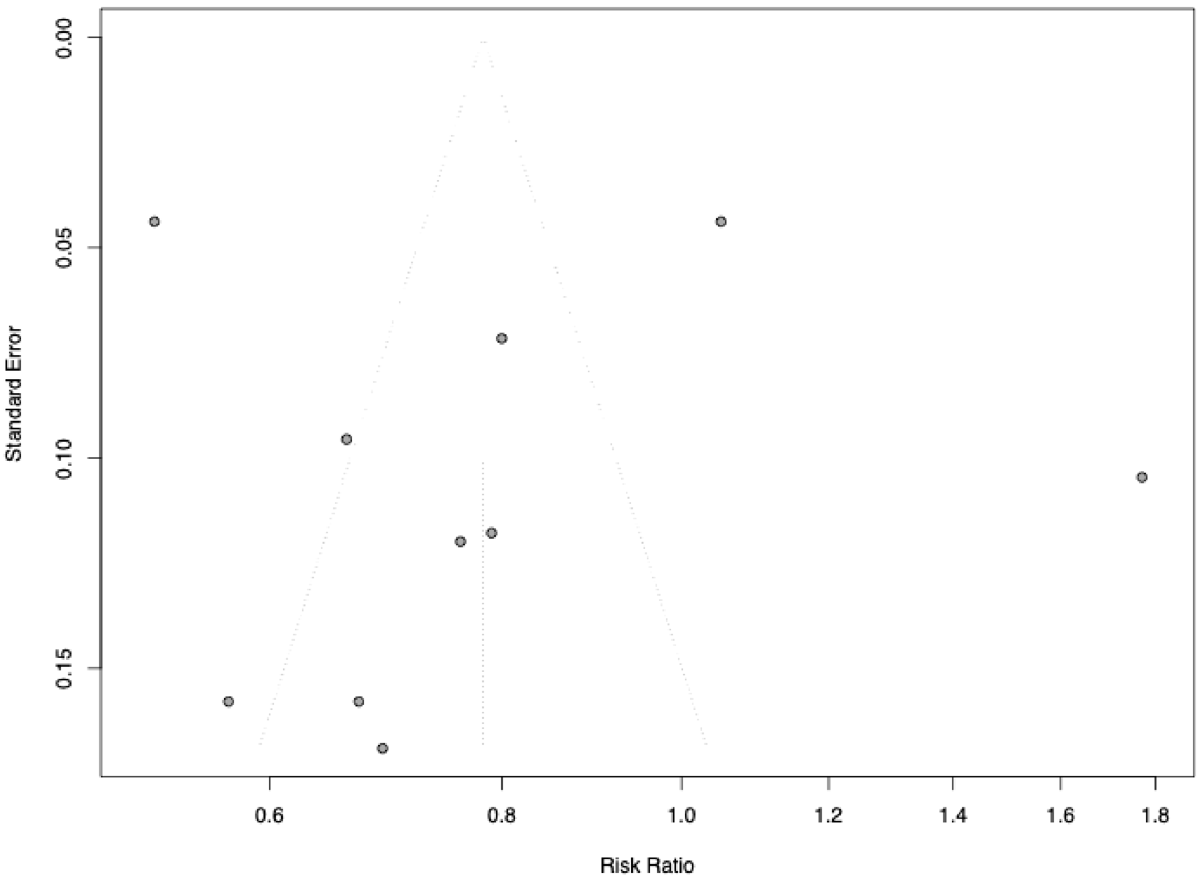

Supplement figure 3: **Funnel plot**

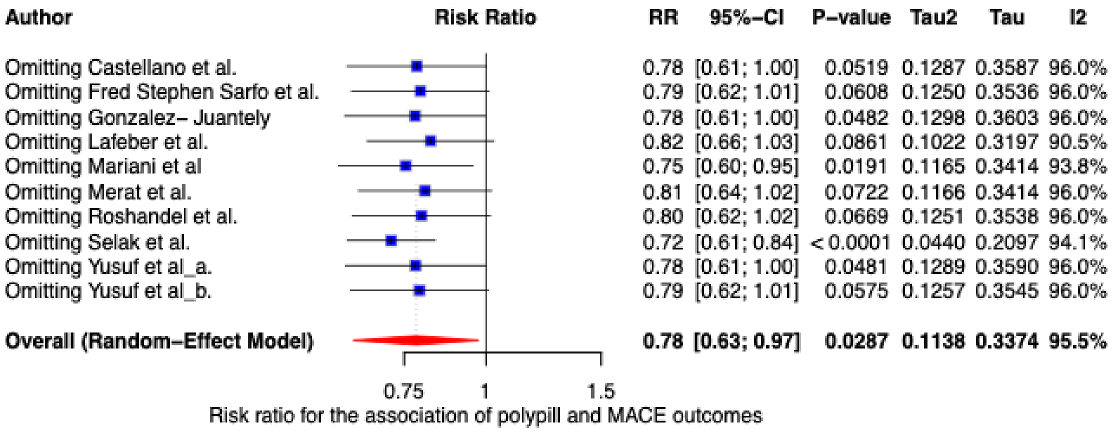

Supplement figure 4: **Influential analysis**
